# Late Incidental Discovery of Compression of the Left Anterior Descending Coronary Artery by an Endocardial Defibrillator Lead

**DOI:** 10.1155/2023/6646715

**Published:** 2023-03-04

**Authors:** Alex Scripcariu, Denis Gaty, Philippe Maury

**Affiliations:** ^1^Department of Cardiology, University Hospital Rangueil, Toulouse, France; ^2^Department of Cardiology, Hospital of Carcassonne, Carcassonne, France; ^3^Unité INSERM U 1048, Toulouse, France

## Abstract

Coronary artery compression/damage by cardiac pacing/defibrillation leads is very rare and often an unknown complication of pacemaker implantation. Here, we present the case of a 71-year-old woman with late discovery of an asymptomatic compression of the left anterior descending (LAD) coronary artery by a defibrillation lead implanted ten years before. This dissuaded us in removing this now malfunctioning lead with high threshold, and an additional right ventricular (RV) lead was implanted along with atrial and left ventricular (LV) leads for allowing resynchronization therapy. Based on the published data, a majority of RV leads are currently implanted in the “anteroseptal area,” which is neighboring the course of the LAD.

## 1. Clinical Case

**Figure 1:**
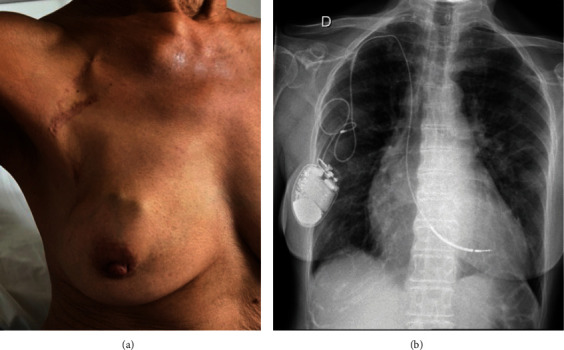
(a) Right thoracic anatomy and (b) anteroposterior chest X-ray.

A 71-year-old woman came to our hospital for aggravated dyspnea and atypical chest pain. Twenty years before, she presented with an anterior wall infarction, which prompted angioplasty of the left anterior descending (LAD). She had been implanted seven years later for primary prevention with a single chamber defibrillator (EVERA S VR DF1, Medtronic, Minneapolis, MN, USA) on the right side connected to a ventricular lead (Saint Jude Medical Durata) with active fixation to the interventricular septum. During follow up, the can glided into the axillary fossa, prompting multiple interventions until a pocket was eventually created by a Plastic Surgeon in the right mammary region (Figure 1).

Device interrogation showed a remaining battery life of 4 years, the absence of any ventricular or supraventricular arrhythmia in the last year, but a dysfunctioning right ventricular (RV) lead with normal detection and impedance but with a high pacing threshold of 5 V at 0.6 ms. The threshold had been steadily increasing over the past years.

**Figure 2:**
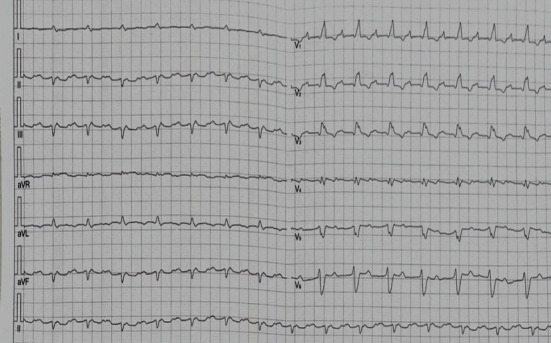
ECG at admission.

The Electrocardiogram (ECG) showed regular sinus rhythm with first degree atrioventricular block, complete right bundle branch block, and left axis deviation with a QRS duration of 180 ms (Figure 2).

Transthoracic echocardiography found a severely altered left ventricle ejection fraction of 20% with a very thin and almost aneurysm-like akinetic area in the apical and anterolateral regions. Minor to moderate functional mitral regurgitation and dyskinesia of the septum were also present.

**Figure 3:**
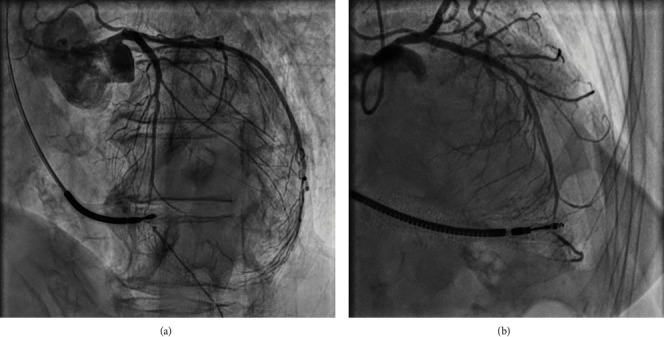
Coronary angiography in the 30° left anterior oblique (a) and 20° right anterior oblique (b) views, showing compression of the LAD. Of note, in the right anterior oblique view, the implantation site is distal to the heart silhouette, suggesting a very anterior position.

As some evolution was suspected concerning her coronary disease, a coronary artery angiography was performed (Figure 3), showing lack of new stenosis, but a compression of the distal LAD coronary artery at the implantation site of the RV lead. Chest pain was not considered to be caused by myocardial ischemia because of the likely lack of viability regarding the thin aneurism like apex, lack of significant Thrombolysis in Myocardial Infarction (TIMI) score alteration, and lack of collaterality. Computed Tomography (CT) scan was not performed due to the high artefacts expected to be caused by the defibrillation lead.

**Figure 4:**
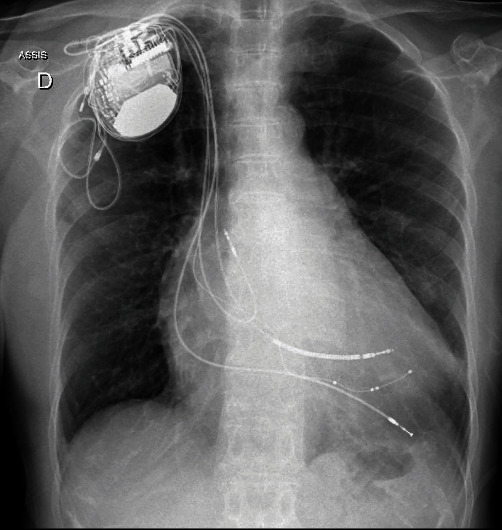
Anteroposterior fluoroscopic view showing the final placement of the leads and of the can.

**Figure 5:**
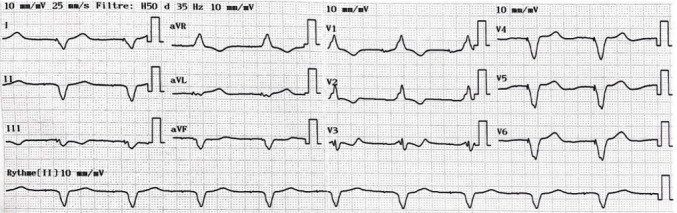
ECG after resynchronization.

As recommended by the European Society of Cardiology (ESC) guidelines [1], the patient underwent an upgrading intervention. A decision was made not to extract the dysfunctioning RV lead because of its old age (15 years since implantation) and its dangerous proximity to the LAD. We proceeded to the implantation of a new IS1 RV lead (Ingevity, Boston Scientific, Marlborough, MA, USA), an IS4 left ventricular (LV) lead (Attain, Medtronic), and an IS4 Atrial lead (Tendril, Saint Jude Medical/Abbott, Abbott Park, IL, USA), leaving in place the ancient Durata DF1 lead (Saint Jude Medical/Abott) for its shocking coil. The choice to not implant a new DF 4 Lead was due to the restricted intravascular space. A new pocket was formed in the proximity of the subclavian region to optimize the defibrillation vector, and the ancient DF 1 lead was tunneled after careful dissection. The new Medtronic CRT-D IS1/DF1 device was thoroughly attached to the pectoral muscle fascia to prevent migration. The following day chest X-ray (Figure 4) showed no lead displacement and the ECG revealed satisfactory resynchronization pattern (Figure 5).

## 2. Discussion

Although device implantation has some potentially fatal complications (see Table 1) [2], procedure-related death is exceptionally rare (0–0.1%).

Damage to the coronary arteries and related structures from pacemaker and implantable cardioverter-defibrillator leads is an even more rarely reported complication, which can lead to myocardial infarction and pericardial tamponade, which can occur acutely or even years later [3]. Incidence of this type of complication is probably very low, and reported cases are exceptional among the literature. Hints could be chest pain that appears close to the intervention and increase of pacing threshold, especially in unipolar configuration [4–6] as for other types of perforation. Usually, management is diverse, and case by case discussion is needed as lead revision is of potential devastating consequence.

There have been identified several factors increasing the risk for lead perforation, such as an older age, a female gender, a Body Mass Index (BMI) <20, higher LV ejection fraction, heart failure, left bundle branch block, previous temporary pacing, small diameter ICD leads, active fixation leads, longer fluoroscopy time, and also factors decreasing the risk, such as BMI >35, previous cardiac surgery, higher implantation procedure rate, diabetes mellitus, and atrial fibrillation [7, 8].

In an imaging study, Pang et al. sought out to better identify the actual incidence of coronary artery damage albeit in a subclinical presentation and to better understand the anatomical relationship between the pacemakers/defibrillators lead implantation sites and the coronary vasculature. In their CT-scan series, leads in the RV antero-septal area were only a few millimeters (median 4.7 mm) away from the LAD, which lies in the anterior interventricular groove. This is due to the fact that implantation is done through fluoroscopy, which is a two-plane imaging method, and leads expected to be in the RV septum in left anterior oblique (LAO) view are actually more at the junction with the RV anterior wall and thus closer to the interventricular groove [9].

The same group suggested a more detailed approach to implantation, describing a technique to better target the true inter-ventricular septum away from the LAD. This would be achieved not only using a leftward shift of the lead in the LAO view but also by using the right anterior oblique view and targeting the middle of the cardiac silhouette together with a paced-QRS duration <140 ms [10].

## Figures and Tables

**Table 1:** Device-related complications.

Procedure-related mortality	0–0.1%
30-day mortality	0–0.6%
Pneumothorax	0.4–2.8%
Clinically relevant perforation	0.1–1.5%
Pericardial effusion	10.2%
Tamponade	0.5–1.5%
Pocket hematoma	0.2–16%
Infection	0.6–3.4%
Lead dislodgement	1.2–3.3%
Other: arrhythmias, pleural effusion, haemothorax, aortic root perforation, lung perforation, pneumopericardium, constrictive pericarditis, air embolism, myocardial infarction, diaphragmatic or intercostal pacing, stroke, brachial plexus palsy, phrenic nerve palsy, acute access vein thrombosis, pulmonary embolism, and tricuspid valve damage	<0.5%

## Data Availability

The data used to support the findings of this study are included within the article. In addition, some data analyzed in this study were a re-analysis of existing data, which are openly available at locations cited in the reference section.
